# A hypocaloric high-protein diet supplemented with β-cryptoxanthin improves non-alcoholic fatty liver disease: a randomized controlled trial

**DOI:** 10.1186/s12876-020-01502-w

**Published:** 2020-10-20

**Authors:** Fatemeh Haidari, Abdollah Hojhabrimanesh, Bizhan Helli, Seyed-Saeed Seyedian, Kambiz Ahmadi-Angali, Behnaz Abiri

**Affiliations:** 1 Department of Nutrition Sciences, Nutrition and Metabolic Diseases Research Center, School of Paramedical Sciences, Ahvaz Jundishapur University of Medical Sciences, 61357-15794 Ahvaz, Iran; 2 Department of Gastroenterology, School of Medicine, Ahvaz Jundishapur University of Medical Sciences, Ahvaz, Iran; 3 Department of Epidemiology and Biostatistics, School of Health, Ahvaz Jundishapur University of Medical Sciences, Ahvaz, Iran; 4 Department of Nutrition, Faculty of Paramedicine, Ahvaz Jundishapur University of Medical Sciences, Ahvaz, Iran

**Keywords:** Non-alcoholic fatty liver disease, β-Cryptoxanthin, High protein diet

## Abstract

**Background:**

Despite promising animal data, there is no randomized controlled trial (RCT) on the effects of high protein (HP)-diet and/or β-cryptoxanthin in non-alcoholic fatty liver disease (NAFLD). Aims: Safety and efficacy assessment of a hypocaloric HP-diet supplemented with β-cryptoxanthin in NAFLD.

**Methods:**

Ninety-two Iranian NAFLD outpatients were recruited for this 12-week, single-center, parallel-group, double-blind RCT and randomized into 4 arms (n = 23): HP-diet and β-cryptoxanthin (hypocaloric HP-diet + β-cryptoxanthin), HP-diet (hypocaloric HP-diet + placebo), β-cryptoxanthin (standard hypocaloric diet + β-cryptoxanthin), and control (standard hypocaloric diet + placebo). Serum levels of liver enzymes and grade of hepatic steatosis were assessed at baseline and study endpoint as outcome measures.

**Results:**

In the intention-to-treat population (N = 92), HP-diet and β-cryptoxanthin group experienced greater 12-week reductions in serum levels of liver enzymes than control group (mean difference for alanine aminotransferase, aspartate aminotransferase, alkaline phosphatase, and gamma-glutamyl transferase: − 27.2, − 7.2, − 39.2, and − 16.3 IU/L, respectively; all *p* < 0.010). Clinical remission rate (achieving grade 0 hepatic steatosis) in HP-diet and β-cryptoxanthin group (82.6%) was also higher than other groups (13.0%, 17.4%, and 0.0% in HP-diet, β-cryptoxanthin, and control groups, respectively; *p* < 0.001). Sixteen patients reported minor adverse events.

**Conclusion:**

A hypocaloric HP-diet supplemented with β-cryptoxanthin safely and efficaciously improves NAFLD.

***Trial registration number*:**

This trial was registered at https://www.irct.ir as IRCT2017060210181N10.

## Background

Non-alcoholic fatty liver disease (NAFLD) is the most common chronic liver disease all over the world. It affects up to 30% of adults in Western countries and 15% in Asian countries and also an elevating number of children [1]. It includes a spectrum of disease from pathological accumulation of triglyceride (TG), steatosis, to an inflammatory response, non-alcoholic steatohepatitis (NASH) and end-stage of liver diseases, specifically cirrhosis and/or hepatocellular carcinoma [2, 3]. NAFLD is becoming an important health challenge globally, not only for its increasing prevalence, but also for its metabolic complications and contributing to substantial morbidity, mortality, and healthcare costs as well as a significant reduction in quality of life [4–9]. Due to the lack of effective pharmacotherapy options, weight reduction through lifestyle modifications encompassing a hypocaloric diet alone or together with increased physical activity remains the first-line of treatment for NAFLD [4, 10, 11].

Since diet is widely recognized as a major contributor to the development and pathogenesis of NAFLD, identification of dietary components capable of preventing or treating this condition is a necessity and could be of significant public health importance [12, 13]. In this respect, the potential beneficial effects of high dietary intakes of protein and carotenoids, particularly β-cryptoxanthin (i.e., a major dietary provitamin A carotenoid largely found in citrus fruits and relatively abundant in human blood and tissues), on NAFLD has recently received lots of attention [12, 14]. In fact, the role of high protein (HP)-diets in improvement of liver enzymes and hepatic steatosis in NAFLD is supported by substantial animal data and a few quasi-experimental studies in humans [15–25]. Similarly, findings of a couple of experimental works in animals suggest that β-cryptoxanthin plays a preventive and even therapeutic role against NAFLD [26, 27].

Despite these promising data, to our knowledge the effects of HP-diet and/or β-cryptoxanthin in NAFLD has never been tested in a randomized controlled trial (RCT), which is considered the gold standard for evidence-based assessment of therapeutic interventions because of its ability to minimize or avoid bias [28]. The present study was thus conducted to assess the safety and efficacy of a hypocaloric HP-diet supplemented with β-cryptoxanthin in NAFLD in a RCT design. It was hypothesized that a hypocaloric HP-diet supplemented with β-cryptoxanthin would lead to more significant improvements in NAFLD, compared to a standard hypocaloric diet.

## Methods

### Patients

In the present study, patients were consecutively recruited by local advertisement from overweight/obese (25 ≤ body mass index [BMI] < 40 kg/m^2^) adult (18 ≤ age < 60 years) volunteers visiting the gastrointestinal outpatient clinic of Golestan Hospital of Ahvaz Jundishapur University of Medical Sciences (AJUMS), Ahvaz, Iran. Eligible NAFLD patients required to have a grade 2–3 (i.e., moderate-to-severe) hepatic steatosis on ultrasonography (US) in the absence of any secondary causes of hepatic steatosis, including excessive alcohol intake (≥ 30 g/day for males and ≥ 20 g/day for females) and other etiologies of liver disease and steatosis.

The study protocol conforms to the ethical guidelines of the 1975 Declaration of Helsinki (6th revision, 2008), as reflected in a priori approval by the AJUMS’s human research committee (reference number: IR.AJUMS.REC.1396.138). Written informed consent was obtained from each patient included in the study. Patient recruitment took place from September 23, 2017 through January 01, 2018, and the last recruited patient completed the final follow-up visit on March 26, 2018. This trial was registered at https://www.irct.ir as IRCT2017060210181N10, in which the full trial protocol can be accessed.

### Study design

**Fig. 1:**
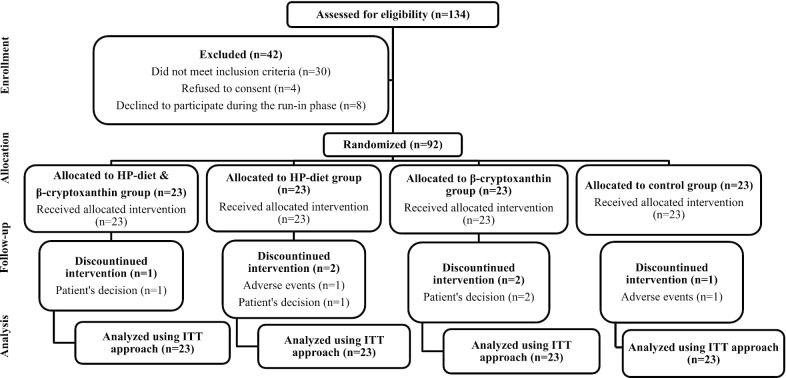
Patient flow diagram throughout the study. *HP *High-protein; *ITT *intention-to-treat

In the present single-center, multi-arm, parallel-group RCT, a total of 100 eligible NAFLD patients were initially studied for a 1-week run-in phase to establish their current energy requirements. Throughout this phase, patients were instructed to provide a 3-days dietary record (2 weekdays and 1 weekend day). Of these, 8 patients declined to participate in the study (Fig. 1), leaving a total of 92 eligible patients (52.2% female; mean age 37.2 years) available for randomization at the end of the run-in phase. Using computer-generated random permuted blocks of size 4 or 8 stratified by gender (male or female) and hepatic steatosis (grade 2 or 3), patients were assigned in a 1:1:1:1 fashion to 4 groups (n = 23) labeled as control, β-cryptoxanthin, HP-diet, and HP-diet and β-cryptoxanthin to receive their corresponding outpatient interventions for 12 weeks. Patients in the HP-diet and β-cryptoxanthin group received a hypocaloric HP-diet supplemented with 6 mg/day of β-cryptoxanthin, while those in the HP-diet group were given the same diet along with a β-cryptoxanthin placebo. On the other hand, those in the β-cryptoxanthin group received a hypocaloric normal protein (NP)-diet with 6 mg/day of β-cryptoxanthin, whereas patients in the control group were given the same diet and a β-cryptoxanthin placebo. The 12-week intervention period of this study was chosen according to a recent work in which an intervention period of > 10 weeks was required for a hypocaloric HP-diet to improve liver enzymes in patients with NAFLD [22].

The random allocation sequence was generated by an independent statistician at the AJUMS School of Health and was given in sequentially numbered, opaque, and sealed envelopes to the study clinician responsible for assessing and enrolling the patients at the Golestan Hospital. After enrollment and completion of all baseline assessments, each patient received a sealed envelope with his/her full name written on it and was referred to an independent dietician at the AJUMS School of Paramedical Sciences who opened the envelopes and assigned patients to their allocated interventions. The random allocation sequence was concealed, and patients, healthcare providers, data collectors, and outcome adjudicators remained blinded to the allocated interventions until the last recruited patient completed the final follow-up visit.

This study adheres to CONSORT guidelines and included a completed CONSORT checklist as an additional file.

### Interventions

The patients’ weight maintenance level of energy intake at baseline was calculated using the standard equations for prediction of total energy expenditure (TEE) in overweight and obese individuals 19 years and older according to their gender, age, weight, height, and physical activity level [29]. The calculated TEE value was then used by an independent dietician to prescribe an individually tailored, food exchange-based, 500 kcal/day (i.e., 2092 kJ/day) deficit hypocaloric diet for each patient. The percent of energy intake from carbohydrate, fat, and protein in diets prescribed to the patients in the control and β-cryptoxanthin groups were approximately 55%, 30%, and 15%, respectively. The corresponding values in diets prescribed to those in the HP-diet and HP-diet and β-cryptoxanthin groups were approximately 45%, 30%, and 25%, respectively. These values were chosen since they are within the acceptable macronutrient distribution ranges presented in the dietary reference intake tables [29], and also because they are easily achievable. In all study groups, animal and plant sources of protein each contributed to about 50% of overall dietary protein intake.

β-Cryptoxanthin powder, containing 1% β-cryptoxanthin and approximately 99% starch, was purchased from the Shanghai Tianfu Chemical Co., Shanghai, China. β-cryptoxanthin powder and pure starch, as β-cryptoxanthin placebo, were then encapsulated in 600 mg, tasteless, odorless, and identical-in-appearance oral capsules at the AJUMS School of Pharmacy. Before randomization, the capsules were packed in identical, opaque, and sealed containers by a person other than the researchers, coded with letters A or B, and handed over to the person responsible for assigning patients to their allocated interventions. Patients were asked to take 1 capsule/day of β-cryptoxanthin or β-cryptoxanthin placebo with a glass of water after dinner. The decision to choose a 6 mg/day dosage of β-cryptoxanthin supplementation in the present work was based on a previous dose-finding study in which this particular dosage was associated with the most significant rise in serum β-cryptoxanthin level without causing any serious adverse events [30].

In addition to the scheduled follow-up visits at week 6 and 12 of the intervention period, weekly phone follow-ups were performed to minimize the drop-out rate. Adverse events and adherence, defined as taking ≥ 90% of prescribed capsules, were recorded at every scheduled follow-up visit.

### Outcome measures

Objective assessment of the primary (i.e., serum levels of liver enzymes and grade of hepatic steatosis) and secondary (i.e., improvement in hepatic steatosis: achieving grade 0 hepatic steatosis) outcome measure was conducted at baseline and study endpoint. Hepatic steatosis was diagnosed via upper abdominal US performed by a trained radiologist at the Golestan Hospital using a Toshiba Nemio 30 ultrasound system (Toshiba Co., Tokyo, Japan) equipped with a 3.5 MHz linear transducer and was classified into 4 grades as follows: grade 0 (no steatosis); grade 1 (mild steatosis); grade 2 (moderate steatosis); and grade 3 (severe steatosis). Fasting serum levels of liver enzymes (IU/L) including alanine aminotransferase (ALT), aspartate aminotransferase (AST), alkaline phosphatase (ALP), and gamma-glutamyl transferase (GGT) were measured via enzymatic methods using commercially available kits (Pars Azmun Co., Tehran, Iran) on an Alcyon 300 automated analyzer (Abbott Laboratories, Abbott Park, IL, USA) by a trained lab technician at the Golestan Hospital on the same day as the blood sampling.

### Other variables

Serum β-cryptoxanthin level (μg/mL) was measured at baseline and week 12 of the intervention period using isocratic reverse-phase high-performance liquid chromatography. The data on demographics including gender (male, female) and age (year) were obtained by a general questionnaire at baseline. Anthropometric variables including weight (kg), height (cm), and waist circumference (WC; cm) were recorded at baseline and every scheduled follow-up visit in accordance to the standard guidelines. BMI (kg/m^2^) was then computed using the following standard formula: BMI = weight (kg)/[height (m)]^2^. In addition to the run-in phase, each patient provided a 3-days dietary record at week 6 and week 12 of the intervention period. The dietary records were then reviewed and analyzed by the Nutritionist IV (First Databank, San Bruno, CA, USA) to determine patients’ daily energy intake (Kcal/day) and percent of energy intake from carbohydrate, fat, and protein ((% of energy). Physical activity (metabolic equivalent-min/week) was assessed at baseline and every scheduled follow-up visit using the valid and reliable Iranian version of the International Physical Activity Questionnaire-Short Form [31–33]. The study clinician at the Golestan Hospital administered the general and physical activity questionnaires via face-to-face interviews, reviewed and analyzed the dietary records, and recorded the anthropometric variables.

### Statistical analysis

The sample size was estimated based on one of the primary outcome measures (i.e. serum ALT level). Using the standard deviations obtained from previous studies [24, 27], a minimum sample size of 18 patients per group was required to detect a minimum difference of 20 IU/L in mean serum ALT level among study groups with a statistical power of 80% and a type-1 error of 5%. However, to allow for a drop-out rate of about 20%, it was finally decided that 23 patients per group would be recruited.

Statistical analyses were performed using the intention-to-treat approach, including all randomized patients (N = 92). The chi-square test or the Fisher’s exact test was used for comparison of categorical variables among study groups as appropriate, while pairwise within group comparison of grade of hepatic steatosis was conducted using the Wilcoxon signed-rank test. For continuous variables, the normality assumption was initially evaluated by the Shapiro–Wilk test, and those with a non-normal distribution were normalized by log-transformation prior to any further analysis. Pairwise within group comparison of continuous variables was then performed using the paired sample T-test. The one-way analysis of variance (ANOVA) was used for comparison of continuous variables among study groups, while the one-way analysis of covariance (ANCOVA) was applied to compute and compare the multivariable adjusted means of continuous outcomes (e.g., serum levels of liver enzymes at study endpoint) among study groups. For each continuous outcome, the corresponding baseline value, gender, age, and 12-week changes in BMI and WC were included as covariates in the ANCOVA. In case of any significant differences, the ANOVA and the ANCOVA were followed by pairwise between group comparisons using the Bonferroni post-hoc test to adequately adjust for multiple comparisons. The IBM SPSS Statistics 21 (IBM Corp., Armonk, NY, USA) was used to conduct all analyses, considering a 2-sided *p* value of < 0.050 as statistically significant. Data were presented as n (%) or mean [95% confidence intervals].

## Results

In this study, overall, 93.5% of all randomized patients completed the study. Figure 1 illustrates the patient flow diagram throughout the study.

The data on drop-out, adherence, and adverse events during the 12-week intervention period are presented in Table 1. There were no significant differences in drop-out, adherence, and adverse events among study groups. In addition, the data on demographics and study outcomes at baseline are shown in Table 2. No significant differences were observed in terms of demographics and study outcomes at baseline among study groups.

**Table 1:** Data on drop-out, adherence, and adverse events during the 12-week intervention period (N = 92)^a,b^

	All	HP-diet and β-cryptoxanthin	HP-diet	β-Cryptoxanthin	Control	*p *value^c^
Drop-out (discontinuing the intervention)	6 (6.5)	1 (4.3)	2 (8.7)	2 (8.7)	1 (4.3)	1.000
Adherence (taking ≥ 90% of prescribed capsules)	84 (91.3)	22 (95.7)	20 (87.0)	21 (91.3)	21 (91.3)	0.956
Adverse events (reporting gastrointestinal discomfort or headache)	16 (17.4)	5 (21.7)	5 (21.7)	3 (13.0)	3 (13.0)	0.803

*HP* high-protein

^a^In each group n = 23

^b^Data are presented as n (%)

^c^Obtained by the Fisher’s exact test

**Table 2:** Data on demographics and study outcomes at baseline (N = 92)^a,b^

	All	HP-diet and β-cryptoxanthin	HP-diet	β-Cryptoxanthin	Control	*p *value^c^
Gender						1.000
Male	44 (47.8)	11 (47.8)	11 (47.8)	11 (47.8)	11 (47.8)	
Female	48 (52.2)	12 (52.2)	12 (52.2)	12 (52.2)	12 (52.2)	
Age (year)	37.2 [35.4; 38.9]	38.4 [34.6; 42.3]	36.4 [32.9; 39.8]	38.1 [34.8; 41.4]	35.6 [31.7; 39.6]	0.626
Serum ALT (IU/L)	70.9 [68.0; 73.7]	71.0 [65.2; 76.7]	70.7 [65.2; 76.2]	71.5 [64.3; 78.7]	70.3 [64.5; 76.1]	0.997
Serum AST (IU/L)	39.1 [36.0; 42.3]	39.3 [33.4; 45.3]	37.5 [30.4; 44.7]	41.0 [34.3; 47.6]	38.6 [31.7; 45.5]	0.779
Serum ALP (IU/L)	193.8 [184.6; 203.1]	194.0 [176.2; 211.8]	190.4 [167.2; 213.7]	194.2 [177.6; 210.7]	196.8 [177.0; 216.6]	0.933
Serum GGT (IU/L)	47.4 [45.6; 49.2]	48.2 [44.4; 51.9]	47.8 [43.8; 51.7]	47.3 [43.9; 50.7]	46.3 [42.3; 50.4]	0.883
Hepatic steatosis						1.000
Grade 0 (no steatosis)	0 (0.0)	0 (0.0)	0 (0.0)	0 (0.0)	0 (0.0)	
Grade 1 (mild steatosis)	0 (0.0)	0 (0.0)	0 (0.0)	0 (0.0)	0 (0.0)	
Grade 2 (moderate steatosis)	48 (52.2)	12 (52.2)	12 (52.2)	12 (52.2)	12 (52.2)	
Grade 3 (severe steatosis)	44 (47.8)	11 (47.8)	11 (47.8)	11 (47.8)	11 (47.8)	

*ALP* alkaline phosphatase, *ALT* alanine aminotransferase, *AST* aspartate aminotransferase, *GGT* gamma-glutamyl transferase, *HP* high-protein

^a^In each group n = 23

^b^Data are presented as n (%) or mean [95% confidence intervals]

^c^Obtained by the chi-square test and the one-way analysis of variance for categorical and continuous variables, respectively

Table 3 presents the data on anthropometrics, dietary intakes, physical activity, and serum β-cryptoxanthin at baseline and study endpoint. There were no significant differences in anthropometrics, dietary intakes, physical activity, and serum β-cryptoxanthin at baseline among study groups. As expected from the study design, percent of energy intake from protein in patients of the HP-diet and β-cryptoxanthin and HP-diet groups at study endpoint was significantly higher than those in the other groups (all *p* < 0.001). Similarly, patients in the HP-diet and β-cryptoxanthin and β-cryptoxanthin groups achieved higher serum β-cryptoxanthin levels at study endpoint, compared to those in the other groups (all *p* < 0.001). Moreover, pairwise within group comparisons revealed significant reduction of energy intake, weight, BMI, and WC in all study groups during the 12-week intervention period (all *p* < 0.001). On average, patients in the HP-diet and β-cryptoxanthin and HP-diet groups had lost 6.8% and 6.9% of their baseline weight at study endpoint respectively, while the corresponding values in the other groups (i.e., 3.9% in both β-cryptoxanthin and control groups) were significantly lower (all *p* < 0.001). No significant within group changes were observed in terms of physical activity in any of study groups. In brief, the values presented in Table 3 indicate the high compliance of patients to the study protocol.

**Table 3:** Data on anthropometrics, dietary intakes, physical activity, and serum β-cryptoxanthin at baseline and study endpoint (N = 92)^a,b,c^

	HP-diet and β-cryptoxanthin	HP-diet	β-Cryptoxanthin	Control	*p *value^d^
Weight (kg)					
Baseline	94.3 [87.3; 101.4]	94.2 [89.2; 99.3]	93.8 [87.8; 99.7]	95.5 [88.1; 102.8]	0.983
Week 12	88.0 [81.1; 95.0]	87.8 [82.7; 92.9]	90.1 [84.3; 95.8]	91.9 [84.3; 99.6]	0.756
*p *value^e^	< 0.001	< 0.001	< 0.001	< 0.001	
BMI (kg/m^2^)					
Baseline	32.8 [31.2; 34.3]	33.9 [32.3; 35.5]	33.2 [31.7; 34.6]	33.1 [31.7; 34.8]	0.758
Week 12	30.5 [29.0; 32.1]	31.6 [30.0; 33.1]	31.9 [30.5; 33.2]	31.8 [30.0; 33.6]	0.576
*p *value^e^	< 0.001	< 0.001	< 0.001	< 0.001	
WC (cm)					
Baseline	104.5 [99.1; 109.9]	104.7 [101.0; 108.5]	103.6 [100.1; 107.1]	104.4 [99.2; 109.5]	0.987
Week 12	97.8 [92.4; 103.1]	97.8 [94.0; 101.6]	99.3 [96.0; 102.5]	100.2 [94.7; 105.8]	0.816
*p *value^e^	< 0.001	< 0.001	< 0.001	< 0.001	
Energy (Mcal/day)					
Baseline	2.9 [2.6; 3.1]	2.8 [2.6; 3.0]	2.8 [2.6; 3.0]	2.9 [2.6; 3.1]	0.970
Week 12	2.4 [2.1; 2.6]	2.3 [2.2; 2.5]	2.3 [2.1; 2.5]	2.4 [2.1; 2.6]	0.973
*p *value^e^	< 0.001	< 0.001	< 0.001	< 0.001	
Carbohydrate (% of energy)					
Baseline	50.6 [48.0; 53.2]	48.9 [46.6; 51.2]	49.3 [46.8; 51.8]	50.0 [47.5; 52.5]	0.753
Week 12	45.6 [44.9; 46.3]	45.3 [44.4; 46.2]	54.2 [53.5; 55.0]	55.3 [54.5; 56.0]	< 0.001
*p *value^e^	0.001	0.008	< 0.001	< 0.001	
Fat (% of energy)					
Baseline	35.4 [33.5; 37.4]	38.2 [36.1; 40.3]	36.9 [34.4; 39.3]	37.0 [34.5; 39.4]	0.414
Week 12	30.0 [29.5; 30.5]	30.0 [29.4; 30.5]	30.6 [30.1; 31.2]	29.2 [28.7; 29.7]	0.001
*p *value^e^	< 0.001	< 0.001	< 0.001	< 0.001	
Protein (% of energy)					
Baseline	14.0 [12.4; 15.5]	12.9 [11.3; 14.5]	13.8 [12.1; 15.5]	13.0 [11.3; 14.7]	0.680
Week 12	24.4 [23.7; 25.1]	24.7 [23.8; 25.6]	15.1 [14.2; 16.0]	15.5 [14.7; 16.3]	< 0.001
*p *value^e^	< 0.001	< 0.001	0.068	0.016	
β-Cryptoxanthin (mg/day)					
Baseline	0.26 [0.23; 0.29]	0.25 [0.23; 0.27]	0.25 [0.23; 0.27]	0.26 [0.23; 0.29]	0.965
Week 12	0.21 [0.18; 0.23]	0.20 [0.18; 0.22]	0.20 [0.18; 0.22]	0.21 [0.18; 0.23]	0.993
*p *value^e^	< 0.001	< 0.001	< 0.001	< 0.001	
Physical activity (MET-min/week)					
Baseline	572.3 [509.2; 635.4]	560.9 [499.6; 622.2]	544.7 [482.6; 606.8]	561.2 [492.8; 629.5]	0.945
Week 12	572.5 [509.9; 635.2]	560.2 [499.0; 621.4]	546.0 [483.9; 608.0]	563.9 [494.2; 633.6]	0.951
*p *value^e^	0.689	0.687	0.502	0.382	
Serum β-cryptoxanthin (μg/mL)					
Baseline	0.16 [0.14; 0.18]	0.16 [0.14; 0.18]	0.15 [0.13; 0.17]	0.16 [0.14; 0.18]	0.943
Week 12	0.28 [0.25; 0.30]	0.16 [0.14; 0.18]	0.26 [0.23; 0.29]	0.16 [0.14; 0.18]	< 0.001
*p *value^e^	< 0.001	0.848	< 0.001	0.773	

*BMI* body mass index, *HP* high-protein, *MET* metabolic equivalent, *WC* waist circumference

^a^In ach group n = 23

^b^Data are presented as mean [95% confidence intervals]

^c^Dietary intakes are determined by averaging the data from 3-days dietary records completed throughout the study

^d^Obtained by the one-way analysis of variance

^e^Obtained by the paired sample T-test

Table 4 shows the data on study outcomes after the 12-week intervention period. Pairwise within group comparisons showed significant reduction of serum levels of liver enzymes and grade of hepatic steatosis in all study groups during the 12-week intervention period (all *p* < 0.010). Using pairwise between group comparisons in the multivariable adjusted ANCOVA models, we found out that patients in the HP-diet and β-cryptoxanthin group experienced greater 12-week reductions in serum levels of ALT, AST, ALP, and GGT than those in the control group (for ALT: mean difference − 27.2 IU/L; for AST: mean difference − 7.2 IU/L; for ALP: mean difference − 39.2 IU/L; and for GGT: mean difference − 16.3 IU/L; all *p* < 0.010). However, compared to those in the control group, patients in the HP-diet group achieved higher reductions in serum levels of ALT and GGT (for ALT: mean difference − 16.6 IU/L; and for GGT: mean difference − 7.5 IU/L; both *p* < 0.050). In addition, patients in the β-cryptoxanthin group only showed a greater 12-week reduction in serum level of ALT, compared to those in the control group (for ALT: mean difference − 9.6 IU/L; *p* = 0.005). In contrast to baseline, there was also a significant difference in terms of hepatic steatosis at study endpoint among groups so that clinical remission rate in the HP-diet and β-cryptoxanthin group was considerably higher than the other groups (82.6% in the HP-diet and β-cryptoxanthin group versus 13.0%, 17.4%, and 0.0% in the HP-diet, β-cryptoxanthin, and control groups, respectively; *p* < 0.001).

**Table 4:** Data on study outcomes after the 12-week intervention period (N = 92)^a,b,c,d,e^

	HP-diet and β-cryptoxanthin	HP-diet	β-Cryptoxanthin	Control	*p *value
Serum ALT (IU/L)					
Unadjusted	25.7 [20.9; 30.5]	36.0 [30.2; 41.8]	42.6 [37.9; 47.4]	51.6 [47.6; 55.6]	< 0.001
Multivariable adjusted	25.1 [21.0; 29.3]	35.7 [31.3; 40.1]	42.7 [38.5; 47.0]	52.4 [48.1; 56.6]	< 0.001
Change in serum ALT (IU/L)					
Unadjusted	− 45.3 [− 50.2; − 40.3]	− 34.6 [− 39.2; − 30.1]	− 28.9 [− 35.0; − 22.8]	− 18.7 [− 23.7; − 13.7]	< 0.001
Multivariable adjusted	− 45.7 [− 49.9; − 41.6]	− 35.2 [− 39.5; − 30.8]	− 28.1 [− 32.4; − 23.9]	− 18.5 [− 22.8; − 14.2]	< 0.001
Serum AST (IU/L)					
Unadjusted	20.0 [17.5; 22.4]	21.6 [19.8; 23.4]	23.1 [21.0; 25.2]	24.7 [20.2; 29.2]	0.121
Multivariable adjusted	18.9 [16.1; 21.7]	20.7 [17.7; 23.6]	23.8 [20.9; 26.7]	26.0 [23.2; 28.9]	0.039
Change in serum AST (IU/L)					
Unadjusted	− 19.4 [− 25.5; − 13.2]	− 15.9 [− 22.6; − 9.2]	− 17.9 [− 24.3; − 11.4]	− 13.9 [− 19.6; − 8.1]	0.603
Multivariable adjusted	− 20.2 [− 23.0; − 17.4]	− 18.4 [− 21.4; − 15.5]	− 15.3 [− 18.2; − 12.4]	− 13.1 [− 16.0; − 10.2]	0.014
Serum ALP (IU/L)					
Unadjusted	136.1 [112.6; 159.5]	142.7 [122.2; 163.3]	167.1 [151.5; 182.7]	180.6 [161.8; 199.3]	0.009
Multivariable adjusted	137.1 [123.3; 150.8]	150.0 [135.5; 164.4]	163.1 [149.1; 177.2]	176.2 [162.1; 190.4]	0.050
Change in serum ALP (IU/L)					
Unadjusted	− 57.9 [− 76.0; − 39.9]	− 47.7 [− 68.5; − 26.9]	− 27.1 [− 35.3; − 18.9]	− 16.2 [− 23.2; − 9.3]	< 0.001
Multivariable adjusted	− 56.8 [− 70.6; − 43.0]	− 43.9 [− 58.3; − 29.4]	− 30.7 [− 44.7; − 16.7]	− 17.6 [− 31.8; − 3.4]	0.005
Serum GGT (IU/L)					
Unadjusted	17.0 [14.3; 19.7]	25.5 [21.8; 29.2]	27.8 [24.4; 31.2]	33.0 [29.8; 36.3]	< 0.001
Multivariable adjusted	16.9 [13.5; 20.2]	25.7 [22.2; 29.2]	27.6 [24.2; 31.0]	33.2 [29.7; 36.6]	< 0.001
Change in serum GGT (IU/L)					
Unadjusted	− 31.2 [− 35.2; − 27.1]	− 22.2 [− 27.6; − 16.8]	− 19.5 [− 24.0; − 15.0]	− 13.3 [− 16.6; − 10.0]	< 0.001
Multivariable adjusted	− 30.5 [− 33.9; − 27.2]	− 21.7 [− 25.2; − 18.2]	− 19.8 [− 23.2; − 16.4]	− 14.2 [− 17.6; − 10.8]	< 0.001
Hepatic steatosis					< 0.001
Grade 0 (no steatosis)	19 (82.6)	3 (13.0)	4 (17.4)	0 (0.0)	
Grade 1 (mild steatosis)	4 (17.4)	18 (78.3)	16 (69.6)	13 (56.5)	
Grade 2 (moderate steatosis)	0 (0.0)	2 (8.7)	3 (13.0)	10 (43.5)	
Grade 3 (severe steatosis)	0 (0.0)	0 (0.0)	0 (0.0)	0 (0.0)	

*ALP* alkaline phosphatase, *ALT* alanine aminotransferase, *AST* aspartate aminotransferase, *GGT* gamma-glutamyl transferase, *HP* high-protein

^a^In ach group n = 23

^b^Data are presented as n (%) or mean [95% confidence intervals]

^c^The one-way analysis of variance and the one-way analysis of covariance were used to compare the unadjusted and multivariable adjusted means of continuous outcomes among study groups, respectively

^d^For each continuous outcome, the corresponding baseline value, gender, age, and 12-week changes in body mass index and waist circumference were included as covariates in the one-way analysis of covariance

^e^The Fisher’s exact test was used for comparison of categorical outcomes among study groups

## Discussion

As far as we are aware, this is the first RCT to examine the potential beneficial effects of HP-diet, β-cryptoxanthin, or both in NAFLD patients. Findings suggest that a hypocaloric HP-diet supplemented with β-cryptoxanthin leads to more significant improvements in liver enzymes and hepatic steatosis in patients with NAFLD over 12 weeks, compared to a standard hypocaloric diet. Moreover, the relatively low rate (i.e., 21.7%) and benign nature (i.e., mild and self-limiting gastrointestinal discomfort or headache) of the reported adverse events as well as the extremely low drop-out rate (i.e., 4.3%) in the HP-diet and β-cryptoxanthin group suggest that this treatment regimen was relatively well-tolerated by the patients with NAFLD.

In this study, patients in the HP-diet and β-cryptoxanthin and HP-diet groups experienced greater reductions in weight than those in the β-cryptoxanthin and control groups at the end of the study. These observations are consistent with the existing evidence indicating that, in a negative energy balance state, HP-diets are more efficacious in weight reduction as compared to NP-diets [34, 35]. Even though the exact mechanisms are not well-determined, this could be justified by the fact that HP-diets are less calorically efficient than NP-diets (i.e., dietary proteins require more energy to be metabolized than other macronutrients do) and lead to more a significant increase in thermogenesis [34, 35].

Because obesity plays a pivotal role in the development and pathogenesis of NAFLD, lifestyle modifications targeted at achieving a weight reduction of at least 3–5% of normal weight represent the first-line of treatment for affected patients [4, 10, 11]. In this respect, findings of a systematic review of 23 interventional studies examining the therapeutic properties of lifestyle interventions (e.g., a hypocaloric diet) in NAFLD indicate that lifestyle modifications leading to weight loss result in significant reduction of circulating liver aminotransferase levels and/or hepatic fat concentration [36]. In line with these findings, following a hypocaloric diet by our patients for 12 weeks led to significant reduction of serum levels of liver enzymes and grade of hepatic steatosis in all study groups. Since patients in the HP-diet and β-cryptoxanthin, HP-diet, β-cryptoxanthin, and control groups respectively lost an average of 6.8%, 6.9%, 3.9%, and 3.9% of their baseline weight in 12 weeks, the observed improvements of NAFLD in all study groups and especially in the control group could thus be largely attributed to the weight-reducing effects of prescribed hypocaloric diets.

As this is the first RCT to assess the effects of HP-diet and/or β-cryptoxanthin in NAFLD, the direct comparison of our findings with those from similar studies is not feasible at the moment. However, greater improvements in NAFLD in the HP-diet and β-cryptoxanthin group as compared to the control group in this study is in line with findings of a few quasi-experimental studies in humans indicating that the prescription of a hypocaloric HP-diet (providing 35–47% of daily energy intake as protein) for 2–11 weeks in NAFLD patients significantly reduces hepatic fat concentration and/or circulating liver enzyme levels [21–24]. Furthermore, our observations are in accordance with a substantial body of evidence from experimental animal research suggesting that HP-diets with or without calorie restriction significantly improve hepatic steatosis in animal models of NAFLD [15–20, 25]. Moreover, results of the present study are supported by those from a couple of experimental works in animals indicating that administration of a normocaloric diet with 0.003% β-cryptoxanthin to mice with diet-induced non-alcoholic steatohepatitis for 12 weeks leads to significant improvement of hepatic steatosis and circulating liver aminotransferase levels [26, 27].

Although the greater efficacy of a hypocaloric HP-diet supplemented with β-cryptoxanthin in improvement of NAFLD as compared to a standard hypocaloric diet in our study could be partly attributed to the higher weight reduction observed in the HP-diet and β-cryptoxanthin group, it is logical to assume that there might be other mechanisms involved. This assumption is in fact supported by the results of a quasi-experimental study by Bezerra-Duarte et al., in which a hypocaloric HP-diet significantly reduced serum levels of liver enzymes in NAFLD patients in the absence of any significant weight reduction [22]. It is noteworthy that a host of potential mechanistic pathways have been proposed to explain the therapeutic properties of HP-diets in NAFLD, among most important of which are induction of lipolysis and lipid utilization as well as suppression of lipogenesis, cell stress, and inflammation in the liver [15, 16]. In addition, a few mechanisms have recently been identified by which β-cryptoxanthin supplementation might exert some beneficial effects in NAFLD [14]. In fact, mechanistic animal studies suggest that β-cryptoxanthin could suppress insulin resistance, oxidative stress, inflammation, and macrophages/Kupffer cells activation, all of which play central roles in the development and/or progression of NAFLD [14, 26, 27]. At last, it is also possible that combination of a hypocaloric HP-diet and β-cryptoxanthin supplementation in the HP-diet and β-cryptoxanthin group of this study have acted synergistically through all or a few of the aforementioned mechanisms to cause more significant improvements in NAFLD.

Some points need to be considered when interpreting the findings of the present study. First, although recent evidence support the diagnostic accuracy and reliability of US for the detection of moderate-to-severe hepatic steatosis as compared to histology, the gold standard method for NAFLD diagnosis and grading is still liver biopsy [4, 5, 37]. Reporting and Data System developed for standardizing interpreting, reporting, and data collection of hepatocellular carcinoma (HCC) describes 5 major features for accurate HCC diagnosis and several ancillary features, some favoring HCC in particular or malignancy in general and others favoring benignity. Untreated hepatic lesions LI-RADS affords 8 unique categories based on imaging appearance on computed tomography and magnetic resonance imaging, which indicate the possibility of HCC or malignancy with or without tumor in vein. Furthermore, LI-RADS defines 4 treatment response categories for treated HCCs after different locoregional therapy. The LI-RADS v2018 is a reliable imaging modality and reporting system that can be routinely performed for standard interpretation of hepatic observation and prediction of HCC and other hepatic malignancy to improve patient care [38, 39]. Second, it must be noted that due to the nature of prescribed diets it was not possible to achieve complete blinding (e.g., patients in the HP-diet and β-cryptoxanthin and HP-diet groups could have detected that they were consuming more amounts of protein than they normally did). Third, even though our ANCOVA models were adjusted for several potential confounders, the probability of residual confounding bias because of unknown or unmeasured confounding variables (e.g., genetic determinants of NAFLD including the palatin-like phospholipase domain-containing 3 or the transmembrane 6 superfamily member 2 gene polymorphism) cannot be entirely ruled out [5].

### Conclusion

In conclusion, findings of the present RCT indicate that a hypocaloric HP-diet supplemented with β-cryptoxanthin safely and efficaciously improves NAFLD. However, future RCTs with high methodological quality are warranted to further confirm these findings.

## Data Availability

The datasets analyzed during the current study are available from the corresponding author on reasonable request.

## Abbreviations

RCT: Randomized controlled trial
HP: High protein
NAFLD: Non-alcoholic fatty liver disease
NASH: Non-alcoholic steatohepatitis
BMI: Body mass index
US: Ultrasonography
NP: Normal protein
TEE: Total energy expenditure
ALT: Alanine aminotransferase
AST: Aspartate aminotransferase
ALP: Alkaline phosphatase
GGT: Gamma-glutamyl transferase
WC: Waist circumference
ANOVA: Analysis of variance
ANCOVA: Analysis of covariance
HCC: Hepatocellular carcinoma
